# Is the association between childhood maltreatment and aggressive behavior mediated by hostile attribution bias in women? A discordant twin and sibling study

**DOI:** 10.1002/ab.21928

**Published:** 2020-08-27

**Authors:** Ada Johansson, Nicola Rötkönen, Patrick Jern

**Affiliations:** ^1^ Department of Psychology, Faculty of Arts, Psychology and Theology Åbo Akademi University Turku Finland

**Keywords:** aggressive behavior, childhood maltreatment, discordant twin design, hostile attribution bias, social information processing

## Abstract

Understanding the mechanisms behind aggressive behavior (AGG) is vital so that effective prevention and intervention strategies can be developed. Maltreated children are hypothesized to be prone to social information processing biases, such as hostile attribution bias (HAB), which, in turn, may increase the likelihood of behaving aggressively. The first aim of the present study was to replicate findings regarding associations between childhood maltreatment (CM), HAB, and aggression in a population‐based sample of Finnish female twins and their sisters (*N* = 2,167). However, these associations might not be causal but instead confounded by familial factors, shared between the variables. The second aim was, thus, to test the associations when potential confounding by familial (genetic or common environmental) effects were controlled for using a multilevel discordant twin and sibling design within (a) 379 pairs of twins (*n*_pairs_ = 239) or siblings (*n*_pairs_ = 140), and (b) within the 131 monozygotic (MZ) twin pairs. Consistent with previous studies, HAB mediated the association between CM and AGG when familial confounding was uncontrolled. No support was found for the mediation when controlling for familial confounding. Between‐pair associations were found between CM and AGG, and between CM and HAB. In addition, within‐pair associations were found between HAB and AGG, and between CM and AGG, however, these were nonsignificant in the discordant MZ analysis, offering the most stringent control of familial confounding. The results indicate the necessity of taking familial confounding into account when investigating the development of AGG.

## 1 INTRODUCTION

Even though aggressive behavior (AGG) is an adaptive response in some instances, it is often accompanied with a multitude of negative consequences, both for victims and the perpetrator (e.g., Krug, Mercy, Dahlberg, & Zwi, 2002). Given its negative consequences, understanding the development of AGG is important so that evidence‐based preventive interventions can be developed (Dodge, 2006).

Several models have been proposed to explain the emergence of AGG from a developmental point of view. One widely used model is the social information processing (SIP) model (Crick & Dodge, 1994). This model aims at explaining the cognitive and emotional processes that lead to AGG in social situations. According to the model, people experience six mental steps when facing social situational cues: (a) encoding of social cues; (b) interpretation and mental representation of the meaning of those cues; (c) formulation of goals; (d) accessing one or more potential responses from memory; (e) evaluation and selection of a response; and (f) enactment to that response. According to the theory, if a bias occurs in any of these six mental steps, it enhances the likelihood of AGG (Crick & Dodge, 1994; De Castro, Veerman, Koops, Bosch, & Monshouwer, 2002). SIP processes related to AGG have been demonstrated in both children (e.g., Dodge & Crick, 1990; Dodge et al., 2015) and adults (e.g., C. A. Bailey & Ostrov, 2008; Matthews & Norris, 2002).

An example of a bias occurring at the second step of the SIP model is hostile attribution bias (HAB; Crick & Dodge, 1994; De Castro et al., 2002). HAB can occur when the information from social cues (such as the actions and underlying motives of others) is interpreted erroneously as hostile (e.g., Dodge & Coie, 1987). In line with SIP theory, individuals who have a biased interpretation of others' actions as hostile have a higher risk for AGG (e.g., De Castro et al., 2002). Studies show that HAB has been positively associated with AGG both in children (e.g., De Castro et al., 2002) and adults (e.g., C. A. Bailey & Ostrov, 2008; Chen, Coccaro, Lee, & Jacobson, 2012; Coccaro, Fanning, Keedy, & Lee, 2016; Epps & Kendall, 1995; Richey, Brown, Fite, & Bortolato, 2016).

Dodge, Bates, and Pettit (1990) hypothesized that maltreated children are at risk for displaying HAB, because they tend to develop biased and inadequate patterns in the processing of social information, and they are therefore more likely to behave aggressively in ambiguous social situations both in childhood and later in life. Indeed, a history of childhood maltreatment (CM) is a risk factor for later AGG (e.g., Cowie, 2015; Fitton, Yu, & Fazel, 2018), and CM has been associated with HAB in children (e.g., Price & Glad, 2003) and adults (e.g., Coccaro, Noblett, & McCloskey, 2009; Richey et al., 2016). Dodge, Pettit, Bates, and Valente (1995) hypothesized that the association between CM and AGG is mediated through biases made in SIP, such as HAB. Previous studies have found support for partial mediation in children (Dodge et al., 1995), and in adults (Taft, Schumm, Marshall, Panuzio, & Holtzworth‐Munroe, 2008). Furthermore, Calvete and Orue (2011) found that SIP processes mediated the relationship between violence exposure (home, school, or community setting) and AGG in adolescents. A study by Richey et al. (2016) showed an indirect effect of CM through instrumental HAB, but not relational HAB, on reactive aggression. In contrast, a study with a sample of 42 men failed to identify HAB as a mediator between emotional CM and aggression (Cowie, 2015).

Even though previous studies have indicated that the association between CM and AGG is partly mediated through HAB, these associations are not necessarily causal. Instead, they could be confounded by underlying familial (genetic or common environmental) factors shared between the variables. For example, a parent who is genetically prone to AGG and HAB, might be more prone to also maltreat or neglect their children, and thus pass on both genes for AGG and HAB to their child as well as a rearing environment characterized by maltreatment and misinterpretations of others' intentions as hostile. Alternatively, a child who has inherited a predisposition to behave aggressively and/or misinterpret social cues as hostile, might be more prone to evoke maltreatment by his or her parents. It is widely acknowledged that genetic influences contribute to AGG (see e.g., Burt, 2009; Veroude et al., 2016), but studies indicate that also phenotypes related to social cognition (Scourfield, Martin, Lewis, & McGuffin, 1999; Warrier et al., 2018), and CM (Pezzoli, Antfolk, Hatoum, & Santtila, 2019; Pittner et al., 2019; South, Schafer, & Ferraro, 2015) are partly under the influence of genes. Genetic influences on the latter constitutes a form of gene–environment correlation (*r*GE), which indicates that the exposure of an individual to an environment, in this case maltreatment, can be influenced by an individual's genetic makeup (Plomin, DeFries, & Loehlin, 1977). Besides genetic confounds, (unmeasured) factors in the family environment common to the variables (CM, HAB, and AGG) could underlie and explain part of their associations, instead of direct causal influences between them.

Twins discordant for exposure of a risk factor, can be used to control for familial confounding. Using twins, the method enables researchers to control for important confounders, namely effects of familial factors, that is, genetic influences or common environmental influences (i.e., environmental factors that make two twins in a pair more alike) that may be shared between the outcome variable and the assumed causal factor, and could not otherwise be controlled for (McGue, Osler, & Christensen, 2010; Vitaro, Brendgen, & Arseneault, 2009). The method can be extended to also include discordant siblings. The rationale for the method lies on the fact that monozygotic (MZ) twins share 100% of their segregating genes while dizygotic (DZ) twins and siblings, on average, share 50%. In addition, twins and siblings reared together share 100% of their common environmental influences (Vitaro et al., 2009). Therefore, twins or siblings in a pair can be used as each other's controls, matched for effects of common environmental influences, and genetic influences (fully controlled for within MZ twin pairs, and 50% in DZ twin or sibling pairs). Thus, if the effect of a factor, such as HAB or CM on AGG, is causal, one would expect that the twin with higher levels of HAB or CM would also show higher levels of AGG, in comparison to their co‐twin or co‐sibling. If the magnitude of the association to aggression is smaller when measured within twin pairs (discordant for CM or HAB), in comparison to uncontrolled estimates, this would indicate that at least part of the association is confounded by underlying familial (genetic or common environmental) influences shared between the outcome and predictor variables. Using multilevel models, it is possible to estimate associations on the between‐families level (i.e., familial confounding) at the same time as estimating within‐pair (WP) associations (Vitaro et al., 2009).

The method has previously been used to investigate whether familial confounding affects associations between different variables. For example, Forsman and Långström (2012) found the association between CM and violent offending to be primarily explained by familial confounding. Jaffee, Caspi, Moffitt, and Taylor (2004) found in a sample of children evidence for the association between CM and antisocial behavior being partly confounded by genetic factors, and partly uniquely environmental and unconfounded. In a similar manner, other studies found that the association between CM and attention deficit hyperactivity disorder (ADHD) symptoms in adults was partly uniquely environmental and unconfounded but also partly confounded by familial influences (Capusan et al., 2016; Dinkler et al., 2017). The relationship between CM and sexual coercion, on the other hand, seemed to be mostly due to environmental processes instead of familial confounding (Forsman, Johansson, Santtila, Sandnabba, & Långström, 2015). Recently, Sypher et al. (2019) analyzed effects of community violence and parenting on the third step of SIP, goal formulation, using a discordant MZ twins design. They found negative associations between positive parenting and hostile social goals in analyses not controlling for familial confounds, as well as within MZ twins, indicating that the association is in part uniquely environmental thus fulfilling one prerequisite for potential causality. For harsh parenting, on the other hand, the positive association to hostile social goals disappeared when analyzed within MZ twin pairs, suggesting familial confounds. These results suggest that applying a discordant twin design to the study of SIP processes can yield informative results. However, the study by Sypher et al. (2019) focused on the SIP process of goal formation and not HAB, did not include measures of AGG, and thus, did not test for mediation.

The present study extends results from previous studies by aiming to replicate the mediation model of an indirect effect of CM on AGG through SIP, specifically the second step process of HAB, in a population‐based sample of Finnish female twins and their sisters. The second aim was to test this mediation model while controlling for possible familial confounding (either genetic or common environmental in origin) using the discordant twin design (a) as extended to also include siblings (379 twin or sibling pairs) and (b) in only MZ‐twin pairs (110 pairs).

Based on previous studies, the following hypotheses were formulated:

(1) CM is positively associated with HAB and AGG.
(2) HAB is positively associated with AGG.
(3) There is an indirect effect of CM on AGG mediated through HAB.
(4) The associations between CM, HAB, and AGG are at least partially confounded by familial factors. That is, we expected there to be significant associations at the between‐pairs (BPs) level, and that the WPs associations would be smaller compared to the base model where familial confounding was uncontrolled.

## 2 MATERIALS AND METHODS

### 2.1 Participants

The final sample consisted of female twins and their sisters (*N* = 2,167, from altogether 1,729 families) from the Finnish population‐based Genetics of Sexuality and Aggression (GSA) project (Johansson et al., 2013). Only women who participated both in the GSA data collection conducted in 2006 (age *M* = 25.52 years, *SD* = 4.97) and a follow‐up conducted in 2013 (age *M* = 33.11 years, *SD* = 5.00) were included. Even though the GSA 2006 sample includes men, this follow‐up in 2013 targeted only women, and, therefore, men did not have data on all relevant variables and were excluded from the present study.

The data collection conducted in 2006 targeted all Finnish‐speaking twins aged 18–33* *years and their siblings of at least 18 years of age, residing in Finland at the time of data collection (for a detailed description of the sample, see Johansson et al., 2013). Overall, 23,577 adults met the criteria described above, of which 11,663 were women. Of these women, 6,200 responded to the survey, yielding a participation rate of 53.2%. Women who indicated their willingness to be contacted again in the future for additional data collections were contacted again in 2013 for a follow‐up (*n* = 5,197). The final response rate for this follow‐up data collection was 41.8% (*n* = 2,173). The research plans for all GSA data collections were accepted by The Ethics Committee of Åbo Akademi University, in accordance with the Helsinki Declaration. At both data collections, written informed consent was obtained from all participants.

Only women who had data available on at least one of the variables of interest were included. The final sample included 544 MZ twin individuals (131 MZ pairs), 497 DZ same‐sex twins (100 DZ pairs), 462 DZ opposite‐sex twins, 37 twins with undefined zygosity (8 pairs), and 627 sisters to twins. The discordant twin/sibling analyses (see steps two and three in the statistical analyses section) were based on data from all 239 complete twin pairs in addition to 28 pairs of siblings and 112 twin‐sibling pairs (a total of 379 pairs). Only one pair per family was included in the analyses. The analyses were then rerun using only MZ twin pairs.

Zygosity was determined by comparing responses to items of physical resemblance (Sarna, Kaprio, Sistonen, & Koskenvuo, 1978), or by a genetically derived zygosity measure for complete twin pairs with genotype information available (for further details, see Johansson et al., 2013). Questionnaire‐based zygosity determination is widely used in twin research and has shown good validity (e.g., Christiansen et al., 2003; Johansson et al., 2013; Sarna et al., 1978).

### 2.2 Measures

#### 2.2.1 CM

The Childhood Trauma Questionnaire Short Form (CTQ‐SF; Bernstein et al., 2003) was used to measure CM. The questionnaire contains 25 items on maltreatment. The CTQ‐SF is divided into five scales: physical abuse, emotional abuse, sexual abuse, physical neglect, and emotional neglect. Each scale contains five items that are scored on a 5‐point Likert scale with the anchors 1 = “*never true*” to 5 = “*very often true*.” CM was measured in the 2006 data collection. A mean score was calculated based on all items measuring CM. The internal consistency was high in the current sample (*α* = .90). The CTQ‐SF has shown good measurement invariance and good criterion validity (Bernstein et al., 2003).

#### 2.2.2 AGG

AGG in adulthood was measured using the physical and the verbal aggression scales of the widely used Buss and Perry Aggression Questionnaire (AQ; Buss & Perry, 1992). These scales contain 14 items (nine on physical aggression, and five on verbal aggression), scored on a 5‐point Likert scale with the anchors 1 = “*Not at all typical*” and 5 = “*Very typical*.” Data on AGG were used from the 2013 follow‐up data collection. A mean score was calculated based on all 14 items. The questionnaire has shown good reliability and validity (Buss & Perry, 1992; Harris, 1997). The internal consistency for the current sample was acceptable (*α* = .78).

#### 2.2.3 HAB

HAB was assessed using the Social Information Processing—Attribution and Emotional Response Questionnaire (SIP‐AEQ; Coccaro et al., 2009). The questionnaire contains eight vignettes depicting socially ambiguous situations toward the participant. After the description of each vignette, the participant is asked to assess four different statements that describe the potential motives behind the action (e.g., “*Why do you think s/he bumped your arm making you spill your coffee*?”): hostile intent (e.g., “*Because s/he wanted to burn me with the hot coffee*”), indirect hostile intent (e.g., “*This person wanted to make me look bad to the customer*”), instrumental nonhostile intent (e.g., “*This person was focused on the meeting*”) and neutral or benign intent (e.g., “*This person did this by accident*”). Each statement is rated on a scale from 1 to 4 (1 meaning “*not likely at all*” and 4 meaning “*very likely*”). Information about HAB was collected in 2013. A mean HAB score was calculated based on ratings of the response options measuring hostile and indirect hostile intent (correlation between these facets was *r* = .76, *p* < .001). The internal consistency for the current sample was good (*α* = .88). The questionnaire has shown both good discriminant and convergent validity and good internal reliability (Coccaro et al., 2009).

### 2.3 Statistical analyses

Descriptive statistics and correlations were computed for the raw composite variables using IBM SPSS Statistics version 24.0 for Windows (IBM Corp., 2016). There was no missing data for HAB or AGG; however, the CM items included some missing data (1.4% or less per item). Missing values were not imputed. For the main analyses, a series of structural equation modeling (SEM) were used, more specifically path analysis, with Mplus version 8 (Muthén & Muthén, 2017) to estimate regressive paths between CM, HAB, and AGG. AGG was regressed on CM (path c') and HAB (path b), and HAB on CM (path a). The indirect path of CM on AGG through HAB (path ab), together with the total effect of CM on AGG (indirect effect plus direct effect) were also estimated. Maximum likelihood estimation was used with robust standard errors, which enables the inclusion of missing values (Yuan & Bentler, 2000). The main analyses were conducted in three steps. First, to get a “base‐model,” the paths were estimated using the entire sample comprising both twins and sisters to twins (*N* = 2,167). Interdependency between members of the same family was taken into account using the “CLUSTER” option; however, familial confounding was not controlled for. Familial confounding was controlled for (in different degrees) in the second and third steps, using multilevel modeling in pairs of twins, siblings, and twin‐sibling pairs in step two, and only MZ twin pairs in step three. Including both MZ twin pairs and DZ or sibling pairs controls for all confounding due to common environmental influences shared between the variables, whereas some of the genetic confoundings will be left uncontrolled for (due to the fact that DZ twins and siblings only share on average 50% of their genetic influences). Only including MZ twins offers the most stringent control of familial confounding.

**Figure 1:**
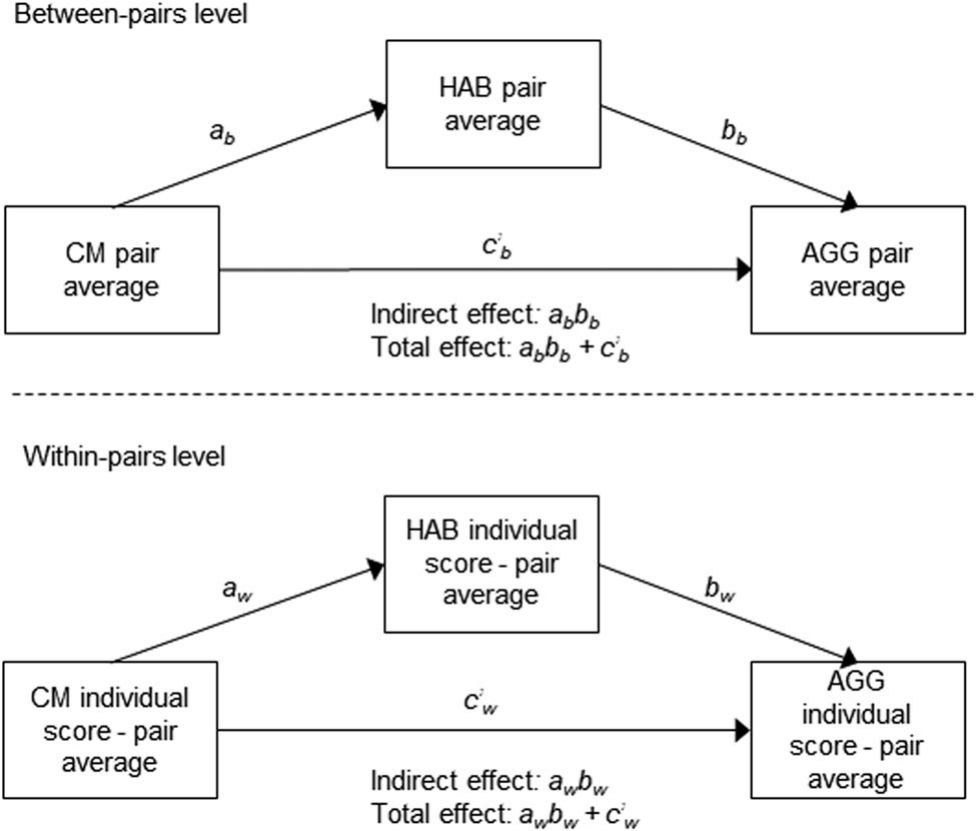
A schematic representation of the discordant twin and sibling multilevel mediational model, in which the variables are decomposed into a between‐pairs (BPs) and a within‐pairs (WPs) part. The mediational model test paths between childhood maltreatment (CM) and hostile attribution bias (HAB) (paths *a*_b_ and *a*_w_), between HAB and aggressive behavior (AGG) (paths *b*_b_ and *b*_w_), as well as direct (paths *c′*_b_ and *c*′_w_), indirect, and total associations between CM and AGG. Single‐headed arrows represent regression paths. Age was included as a covariate, likewise decomposed to a BPs and a WPs part (not depicted)

Multilevel modeling can be used in discordant twin and sibling analyses to model the two levels of nesting: Level 1 is the individual WP level and Level 2 the pair level (i.e., BP associations; Vitaro et al., 2009). All three variables (CM, HAB, and AGG), as well as the covariate age, were decomposed into two variables, respectively; one indicating the WP (Level 1), and the other indicating the BP (Level 2) sources of variance. The pair averages (i.e., the mean score across both individuals in a pair) for respective variables constituted the BP level indicators, and these variables were then grand mean‐centered. The WP indicators were group mean‐centered, which means that the WP indicators were an individual's score on a trait minus the pair average for that specific trait (comparing an individual against her co‐pair). This way, the BP associations reflect familial confounds, that is, genetic and/or common environmental factors contributing to the associations between the variables of interest. The WP associations, on the other hand, reflect unique environmental associations unconfounded (fully within MZ twins and partially within DZ twins or siblings) by underlying familial effects. A schematic model of the multilevel meditational model is depicted in Figure 1. For the discordant twin and sibling analyses, two‐level unconflated multilevel models incorporating mediation (Preacher, Zyphur, & Zhang, 2010) were estimated using Mplus (v. 8). Data were structured in a long format. Again, maximum likelihood estimation with robust standard errors was used. Results from standardized two‐tailed tests were reported including 95% confidence intervals for the regression coefficients.

## 3 RESULTS

### 3.1 Descriptive statistics and correlations

The overall levels of CM, HAB, and AGG were relatively low in the sample (Table 1). As the composite scores of CM and AGG were non‐normally distributed, they were log‐transformed before the remaining analyses. The composite score of HAB was normally distributed and therefore not log‐transformed. As expected, all variables correlated positively with each other (Table 1). The intraclass correlation coefficients (ICCs) indicated that there was WP variability. In addition, the larger ICCs for MZ pairs (genetic correlation 1.0) than for DZ twins and siblings (genetic correlation of 0.5) suggest that genetic factors influence all three traits to some degree.

**Table 1:** Descriptives (means, *SD*s, and ranges), intercorrelations, and ICCs for CM, HAB, and AGG

			Range		Pearson correlations		ICCs [95% CI]		
Variable	*M*	*SD*	Min.	Max.	1.	2.	All pairs	Pairs with *r*_g_ = 1.0	Pairs with *r*_g_ = .5
1. CM	1.42	0.44	1.00	3.92			.51*** [0.44, 0.58]	.69*** [0.59, 0.77]	.42*** [0.31, 0.52]
2. HAB	1.86	0.41	1.00	3.94	.15**		.23*** [0.13, 0.32]	.36*** [0.21, 0.50]	.16** [0.04, 0.28]
3. AGG	1.82	0.43	1.00	4.57	.21**	.21**	.28*** [0.19, 0.37]	.39*** [0.24, 0.53]	.19** [0.07, 0.31]

*Note*: Scale range for HAB is 1–4, and 1–5 for CM and AGG, with higher scores indicating higher levels of the phenomena. Descriptives were based on raw variables but CM and AGG were log‐transformed before the calculation of Pearson and ICCs. *r*_g_ = genetic correlation coefficient (1.0 for MZ‐twin pairs, and 0.5 for DZ‐twin pairs and sibling pairs).

Abbreviations: AGG, aggressive behavior; CI, confidence interval; CM, childhood maltreatment; DZ, dizygotic; HAB, hostile attribution bias; ICC, intraclass correlations coefficient; *M*, mean; MZ, monozygotic; *SD*, standard deviation.

** *p* < .01 level.

*** *p* < .001 level.

### 3.2 Mediation model of the association between CM and AGG through HAB

In the base model when familial confounding was left uncontrolled, significant positive associations of small‐to‐moderate size were found between CM and HAB, as well as HAB and AGG (Table 2). In addition, both the direct effect of CM on AGG, as well as the indirect effect mediated through HAB, were positive and significant, with the indirect effect being considerably smaller in size.

**Table 2:** Standardized path estimates (with 95% confidence intervals) and standard errors of the mediation model in the base model (full sample) as well as in the multilevel discordant twin and sibling analyses

	Base model^a^ (*N* = 2,167)			All pairs^b^ (*N* = 758)			MZ pairs^b^ (*N* = 262)		
	*β* [95% Cl]	*SE*	*p*	*β* [95% Cl]	*SE*	*p*	*β* [95% Cl]	*SE*	*p*
HAB on CM	.15 [0.10, 0.19]	0.02	<.001						
WP				.05 [−0.05, 0.16]	0.06	.33	.03 [−0.13, 0.20]	0.09	.70
BP				.81 [0.13, 0.35]	0.21	<.001	.22 [−0.01, 0.45]	0.12	.06
AGG on HAB	.18 [0.14, 0.23]	0.02	<.001						
WP				.18 [0.08, 0.28]	0.05	<.001	.08 [−0.08, 0.24]	0.08	.32
BP				.02 [−0.02, 0.19]	0.01	.12	.02 [−0.15, 0.19]	0.09	.79
AGG on CM (direct effect)	.19 [0.15, 0.24]	0.02	<.001						
WP				.11 [0.01, 0.20]	0.05	.03	−.08 [−0.23, 0.07]	0.08	.27
BP				.22 [0.15, 0.40]	0.05	<.001	.26 [0.03, 0.49]	0.12	.03
AGG on CM (indirect effect)	.03 [0.02, 0.04]	0.01	<.001						
WP				.01 [−0.01, 0.03]	0.01	.36	.00 [−0.01, 0.02]	0.01	.73
BP				.02 [−0.01, 0.05]	0.01	.14	.01 [−0.03, 0.04]	0.02	.79
AGG on CM (total effect)	.22 [0.17, 0.27]	0.02	<.001						
WP				.12 [0.02, 0.22]	0.05	.02	−0.08 [−0.23, 0.07]	0.08	.29
BP				.29 [0.18, 0.41]	0.06	<.001	.26 [0.05, 0.47]	0.11	.02

*Note*: Age was included as a covariate in the estimation of within‐pairs parameters (except for the MZ analyses since individuals in an MZ pair do not differ in age), and the pair average in age was included as a covariate in estimation of the between‐pairs parameters (results not shown).

Abbreviations: AGG, aggressive behavior; BP, between‐pair; CM, childhood maltreatment; HAB, hostile attribution bias; MZ, monozygotic; SE, standard error; WP, within‐pair.

a The base model did not correct for familial confounding (i.e., it did not separate between within and between parameters) but took family clustering into account.

b Unconflated multilevel mediation models separating between WP and BP associations as analyzed either by including all pairs (not differentiating between genetic relatedness), or only including MZ pairs.

In steps two and three, unconflated multilevel mediation models were used to distinguish between WP associations and BP associations (Table 2). In step two all pairs were included in the analyses (regardless of genetic relatedness being either 100% or 50%). Although both the WP and BP associations between CM and AGG were significant (total effects), the BPs association was stronger in size, indicating familial confounding but also a small partially unconfounded WP association. In other words, pairs with a higher average of CM also had higher pair‐average levels of AGG, in comparison to other pairs. In addition, within pairs, the individual with higher levels of CM, also showed higher levels of AGG in relation to her co‐pair. When separating between the direct and indirect associations, the direct association between CM and aggression was again stronger on the familial level, but significant on both levels (WP and BP), whereas the indirect effects were nonsignificant.

No evidence was seen for a WPs association between CM and HAB; however, the BPs association was significant indicating familial confounding (Table 2). On the contrary, whereas there was no BPs effect for the association between HAB and AGG, the WPs association was significant, indicating that when controlling for partial familial confounding, the individual with higher levels on HAB showed higher levels on AGG, in comparison to her co‐pair.

In step three, only pairs of MZ twins were included in the analyses, controlling for 100% of confounding due to genetic and common environmental influences (Table 2). In these analyses, only the total and direct BP associations between CM and AGG were significant, indicating familial confounding in these associations. None of the remaining associations were significant.

## 4 DISCUSSION

One widely used theoretical model of the development of AGG is the SIP model (Crick & Dodge, 1994). The model posits that people process social information in six different steps and that biases occurring at any of these steps, such as HAB occurring at step two, increase the likelihood of an aggressive response. In addition, according to Dodge et al. (1995), maltreated children are prone to different biases in this SIP model, which in turn, may increase the likelihood of AGG. The aims of the present study were twofold. First, using a large population‐based sample of women, we aimed at replicating previously reported positive associations between CM, HAB, and AGG, and an indirect effect of CM on AGG through HAB (e.g., Calvete & Orue, 2011; Dodge et al., 1995; Richey et al., 2016). Our second aim was to test the mediation model while controlling for familial confounders using the discordant twin method also extended to include siblings.

Consistent with previous findings (e.g., Fitton et al., 2018), we found a positive association between CM and AGG when not controlling for familial confounding. Similarly, we also found a positive association between CM and HAB as previously reported (e.g., Price & Glad, 2003; Richey et al., 2016), confirming our first hypothesis. Also consistent with previous findings (e.g., C. A. Bailey & Ostrov, 2008; De Castro et al., 2002; Richey et al., 2016; Walters, 2007), HAB and AGG were positively associated, confirming the second hypothesis. Furthermore, our results indicated a positive indirect effect of CM on AGG mediated through HAB, confirming our third hypothesis. These results are consistent with previous findings indicating that biases in SIP mediate associations between CM (Dodge et al., 1995; Richey et al., 2016; Taft et al., 2008) or overall exposure to violence (Calvete & Orue, 2011), and AGG. Richey et al. (2016) found in their study that instrumental HAB partially mediated the association between CM and reactive aggression, whereas relational HAB did not. The authors thus hypothesized that some forms of HAB might be more pronounced than other forms in explaining AGG in maltreated individuals (Richey et al., 2016). In the present study, we did not analyze different subtypes of HAB, and the results by Richey et al. (2016) have yet to be replicated. In addition, a study by Cowie (2015) failed to identify HAB as a mediator between emotional CM and AGG; however, this study was likely underpowered (*N* = 42). Mediation effects aside, a study by Chen et al. (2012) indicated that CM could moderate relationships between different aspects of SIP and aggression. With regard to our results, it should also be mentioned that CM had a direct association with AGG, indicating that CM has a unique contribution to AGG, over and above the indirect path through HAB. Besides HAB, problems with emotion regulation or other aspects of SIP like encoding errors, positive evaluation of aggression, or goal formation could act as potential additional mediators (Dodge et al., 1995; Lee & Hoaken, 2007; Sypher et al., 2019). In summary, when familial confounding was not accounted for, we were able to replicate findings from most previous studies indicating positive associations between the variables as well as an indirect effect of CM on AGG through HAB.

Our second aim was to test the mediation model when accounting for confounding by shared underlying familial factors by using an extended discordant twin/sibling design. This was done in two steps using multilevel models, first, by including all twin pairs as well as pairs of siblings, and second, by only including MZ twin pairs. The first step, including all twin and sibling pairs, had the benefit of increased statistical power and thus likely yielded more robust estimates; however, it only offers partial control for genetic confounding. The discordant MZ twin analysis on one hand, fully controlled for familial confounding, but on the other hand, it offered reduced statistical power. These differing strengths and limitations should be kept in mind when interpreting the results.

The multilevel model, including twins and siblings, indicated evidence for familial confounding in the associations between CM and AGG (both the direct and total associations), as well as between CM and HAB, in line with our fourth hypothesis. The indirect effect of CM through HAB was miniscule, and nonsignificant both on the family level as well as within pairs. Therefore, even though we found support for the mediation model in the base model, no significant effects could be detected when the indirect effect was separated into a BPs component and an unconfounded WPs component. These findings are in line with previous studies, which have found genetic or environmental confounding in the associations between CM and other variables. For example, Forsman and Långström (2012) found familial confounding in the association between CM and adult violent offending, and Young‐Wolff, Kendler, Ericson, and Prescott (2011) found confounding by common environmental effects in the association between CM and alcoholism. Similar findings were also shown in a study by Capusan et al. (2016), where the association between CM and ADHD symptoms was partly confounded by genes and environment.

Common environmental influences shared between CM and AGG, or CM and HAB, could be, for example, factors in the family environment such as harsh parenting, or influences of the neighborhood environment or socioeconomic status affecting the entire family. In line with this, a recent discordant twin study did not find support for a unique environmental effect (a prerequisite for a potentially causal influence) of harsh parenting on hostile goal formation, another process of SIP (Sypher et al., 2019), suggesting that harsh parenting could be a potential confounding factor at the family level. On the contrary, no evidence of confounding was seen for the protective association between positive parenting and hostile goal formation (Sypher et al., 2019). Genetic confounding could occur, for example, if parents who are genetically prone to AGG and HAB, are also more likely to maltreat or neglect their children and expose them to a rearing environment characterized by hostility (due to their genetic predispositions). Inferences regarding the nature of the confounder (i.e., whether it is of genetic or common environmental origin) cannot, however, be drawn from the results of the present study. The next step for future research could be to use multivariate twin models to estimate how much of the co‐occurrence of CM, HAB, and AGG is due to genetic and environmental effects shared between the variables.

In addition to the familial confounding discussed above, CM showed a smaller but significant WP association with AGG (the direct and total effects, not the indirect effect). This suggests that the association between CM and AGG is partly confounded by familial factors but may also partly be uniquely environmental. This is in line with studies finding support for partial familial confounding and partial unique environmental influences for the association between CM and antisocial behavior (Jaffee et al., 2004), and between CM and ADHD (Capusan et al., 2016; Dinkler et al., 2017). It is important to bear in mind that the analysis including twins and siblings, did not control for all possible genetic confounding between the variables and might thus yield an underestimation of familial confounding. No evidence of any WP associations was seen in the MZ‐twin analyses, controlling for all common environmental and genetic confounding between the variables.

Contrary to our fourth hypothesis, no familial confounding was evidenced for the association between HAB and AGG. Instead, while controlling for familial effects, the individual with higher levels of HAB showed higher levels of AGG in comparison to her co‐pair, indicative of a unique environmental association (i.e., a significant WP association). This finding is in line with experimental studies suggesting that interventions focusing on modification of SIP processes, could potentially reduce AGG (Penton‐Voak et al., 2013; however see also Hiemstra, Orobio de Castro, & Thomas, 2019). It should be noted, however, that the WPs association between HAB and AGG was nonsignificant and smaller in the more stringent MZ analyses, suggesting that HAB and AGG could still share part of their underlying genetic influences.

Previous research indicates that negatively biased social cognitive factors partially mediate the association between social‐environmental risk factors (such as social rejection and community violence exposure) and AGG (Bradshaw, 2004; Dodge et al., 2003). In other words, the relationship between HAB and AGG could be also be influenced by social‐environmental risk factors outside the family environment (and possibly not shared between twins). Recently, the effect of indirect exposure to community violence on SIP (goal formation) was shown not to be confounded by genetic or common environmental factors (Sypher et al., 2019), suggesting another potential route for the development of HAB.

A strength of the current study was the use of a population‐based sample, and that the sample consisted of twins (and their sisters), allowing for multilevel analyses separating between WP and BP associations. The response rates for the data collections were 53% and 42%, respectively, which are comparable to the response rates of other sexuality‐related surveys (e.g., J. M. Bailey, Dunne, & Martin, 2000; Långström & Zucker, 2005), also taking into account the trend of declining response rates to population‐based surveys (National Research Council, 2013). The sample was at first wave comparable to other population‐based samples on different characteristics such as mean age at first intercourse, rates of sexual abuse, and level of education of the participant's parents (Johansson et al., 2013). There was also no difference in the levels of CM, Wald *χ* ^2^ = 1.17, *df* = 1, *p* = .28, or AGG, Wald *χ* ^2^ = 2.36, *df* = 1, *p* = .12, as measured at the first wave in 2006, between women who participated in the follow‐up in 2013 and those who did not. In addition, studies indicate that results from twin studies can be generalized to the non‐twin population (e.g., Andrew et al., 2001; Johnson, Krueger, Bouchard, & McGue, 2002).

Because the sample included only women, it is unclear to what extent the results are generalizable to men. Gender differences have been reported for both AGG (e.g., Card, Stucky, Sawalani, & Little, 2008), CM (e.g., Stoltenborgh, Van Ijzendoorn, Euser, & Bakermans‐Kranenburg, 2011) and for the association between HAB and aggression (Cillessen, Lansu, & Van den Berg, 2014; De Castro et al., 2002). Thus, it is possible that the relevance of different types of CM experiences in the formation of HAB, as well as associations between maltreatment, HAB, and aggression, might differ between the genders.

The discordant twin and sibling method does not guarantee that any detected WP associations would be causal, even though the method can be used to reject causality; unmeasured factors that led to differences in exposure could also explain differences in the outcome (McGue et al., 2010). In addition, to rule out reverse causality, longitudinal data are needed. In the current study, CM was measured in 2006, whereas AGG and HAB were both measured at a later wave in 2013. Therefore, inferences about the directionality of the associations between CM and later HAB and AGG can be made, but not between HAB and AGG.

Finally, self‐reports were used for all variables, and CM was measured in retrospect. Even though retrospective self‐reports might not be entirely reliable, for example, due to recall biases, a review by Hardt and Rutter (2004), found that retrospective reports of adults on their negative events in childhood can be seen as valid and that people tend to underestimate the occurrence of abuse/neglect more likely than overestimate.

In conclusion, the discordant twin and sibling analyses found no support for the indirect effect of CM through HAB on AGG. The results indicated that the association between CM and AGG, as well as between CM and HAB were confounded by familial influences. In addition, the results suggested that a unique environmental association might exist between HAB and AGG, as well as between CM and AGG. The results indicate the importance of taking familial confounding into account when investigating the development of AGG. Future research should investigate further the nature of overlap in genetic and environmental effects between CM, HAB, and AGG.

## CONFLICT OF INTERESTS

The authors declare that there are no conflict of interests.

## Data Availability

The data that support the findings of this study are available on request from the corresponding author. The data are not publicly available due to privacy or ethical restrictions.
